# Large Volume Electron Microscopy and Neural Microcircuit Analysis

**DOI:** 10.3389/fncir.2018.00098

**Published:** 2018-11-12

**Authors:** Yoshiyuki Kubota, Jaerin Sohn, Yasuo Kawaguchi

**Affiliations:** ^1^Division of Cerebral Circuitry, National Institute for Physiological Sciences (NIPS), Okazaki, Japan; ^2^Department of Physiological Sciences, The Graduate University for Advanced Studies (SOKENDAI), Okazaki, Japan; ^3^Research Fellow of Japan Society for the Promotion of Science (JSPS), Tokyo, Japan

**Keywords:** volume electron microscopy, carbon nanotube, synapse, connectome, ATUM, FIB-SEM, SBEM, segmentation

## Abstract

One recent technical innovation in neuroscience is microcircuit analysis using three-dimensional reconstructions of neural elements with a large volume Electron microscopy (EM) data set. Large-scale data sets are acquired with newly-developed electron microscope systems such as automated tape-collecting ultramicrotomy (ATUM) with scanning EM (SEM), serial block-face EM (SBEM) and focused ion beam-SEM (FIB-SEM). Currently, projects are also underway to develop computer applications for the registration and segmentation of the serially-captured electron micrographs that are suitable for analyzing large volume EM data sets thoroughly and efficiently. The analysis of large volume data sets can bring innovative research results. These recently available techniques promote our understanding of the functional architecture of the brain.

## Introduction

Electron microscopy (EM) has been used in neuroscience research for more than 60 years. EM was introduced as a cutting edge tool to observe a neural structure at super high resolution in a dimension completely different from the optical light microscope, making synaptic structure visible, and it provided unprecedented datasets and an entirely new perspective in neuroscience research. In the 1980s, with Golgi’s silver staining, which sparsely stains individual neurons, or with an immunohistochemical staining method, which targets a specific brain cell population, it has been possible to analyze the ultrastructure, including synaptic contacts, of identified neural structures (Somogyi, 1977; Somogyi and Cowey, 1981; Kubota et al., 1987; Kisvárday et al., 1990). Since the early 1990s, neuronal structures have been three-dimensionally reconstructed from successive electron micrographs captured with manually-collected serial ultrathin sections (Harris et al., 1992; White et al., 1994; Kubota et al., 2007). This method allows us to obtain quantitative information from neural structure, e.g., synapse density, dendritic dimensions and organelle structure. This strategy, however, requires expert skills to cut and collect continuous ultrathin sections, and capturing serial section electron micrographs using transmission EM (TEM) is time-consuming and labor-intensive. Despite these drawbacks, the method attracted neuroscience researchers due to its capability to visualize synaptic contacts between neurons.

In the past decade, new automated/semi-automated systems for the acquisition of serial electron micrographs have been developed and adapted for neuroscience research. These include focused ion beam-scanning EM (FIB-SEM; Heymann et al., 2006; Knott et al., 2008; Merchán-Pérez et al., 2009; Morales et al., 2011; Sonomura et al., 2013; Bosch et al., 2015; Villa et al., 2016; Takemura et al., 2017b; Xu et al., 2017), serial block-face EM (SBEM; Denk and Horstmann, 2004; Helmstaedter et al., 2013; Mikula and Denk, 2015; Schmidt et al., 2017), automated tape-collecting ultramicrotomy (ATUM) with SEM (Hayworth et al., 2006, 2014; Terasaki et al., 2013; Tomassy et al., 2014; Kasthuri et al., 2015), TEM camera array (TEMCA; Bock et al., 2011; Lee et al., 2016; Zheng et al., 2018) and transmission-mode SEM (Kuwajima et al., 2013), in addition to conventional EM using ultra microtomes with TEM (Kubota et al., 2011, 2015; Dufour et al., 2016; Ryan et al., 2016; Bopp et al., 2017; Bloss et al., 2018; Bromer et al., 2018). Each of these methods has unique advantages and drawbacks (Briggman and Bock, 2012; Kubota, 2015). In this review, we will review these different methods and tools for volume EM and expected outcomes, with a focus especially on the ATUM-SEM system, which has the advantage of imaging large regions at high resolution.

## ATUM-SEM

**Figure 1:**
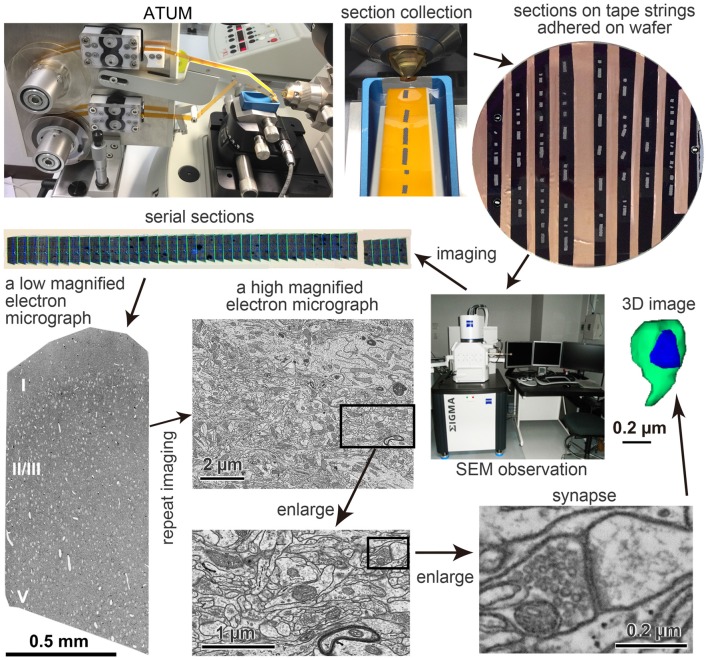
Pipeline of the automated tape-collecting ultramicrotomy (ATUM)-scanning electron microscopy (SEM) method. The pathway of a Kapton tape (orange color) in the ATUM (Boeckeler Instruments, Inc., Tucson, AZ, USA) is shown in the upper left panel. Sections on tape strings adhered on 4-inch wafer is shown in the upper right. The carbon nanotube (CNT) tape strips in the wafer appear in black, which is a color of a conductive double-sided adhesive tape. Serial sections (gray color) are irregularly clustered on the tape. Copper foil tape between the CNT tape was used to electrically ground the CNT layer to the wafer to secure an escape route for incident electrons. The electron micrographs in lower panels are taken from an ultrathin section of the brain tissue on the CNT tape. The left micrograph was taken with a backscattered electron (BSE) detector of field emission SEM (FE-SEM), Sigma (Carl Zeiss Microscopy GmbH, Oberkochen, Germany) and a large area imaging apparatus for SEM, Atlas 5 (Fibics incorporated, Ottawa, ON, Canada), while middle and right panels were taken with an acceleration voltage 1.5 keV, OnPoint BSE detector (Gatan Inc., Pleasanton, CA, USA) in an FE-SEM, GeminiSEM 300 (Carl Zeiss Microscopy GmbH, Oberkochen, Germany). Adapted from Kubota and Kawaguchi (2018) and Kubota et al. (2018).

ATUM-SEM has several valuable features for neural microcircuit investigation (Heymann et al., 2006; Mikuni et al., 2016; Morgan et al., 2016). One of its advantages is that the same serial ultrathin sections can be observed multiple times (Hayworth et al., 2014). We took advantage of this property to image a region of interest (Figure 1) as follows. First, serial sections were imaged at very low magnification (100–200 nm/pixel) to identify all sections on the tape strips attached to a wafer. Then, an electron micrograph of an entire or partial ultrathin section at low magnification (40–50 nm/pixel), where neuronal somata and dendrites can be easily identified, was taken from each of all the sections. A mosaic electron micrograph stitched with 3 × 5 tile images of 8,000 × 8,000 pixels in low magnification covered rat cerebral cortex tissue sections from the pia to the deep layers. The mosaic images were stitched with an image analysis application, Fiji/TrakEM2^1^ (Cardona et al., 2012; Schindelin et al., 2012). Finally, a region of interest in the low-magnified electron micrograph was imaged again from each section, this time at a high magnification (4 nm/pixel) for the analysis of fine neural structure. Thus, we captured electron micrographs of 25,000 × 25,000 pixels covering a large area, i.e., 100 μm × 100 μm, which has a sufficient resolution to see synaptic structures (Figure 1). The serial electron micrographs were aligned using Fiji plugins (Registration/Register Virtual Stack Slices or TrakEM2). We reconstructed neuronal elements in the EM data set three-dimensionally (Figure 1). Using the ATUM procedures, Morgan et al. (2016) obtained a four by four montage of image tiles (each tile was a 25,600 × 25,600 pixel images, 4 nm pixels) from about 10,000 of 30 nm thick serial sections and the final image size was 100 TB of approximately 400 μm square × 280 μm. It took about 10 days to acquire images with in lens secondary electron (SE) detector and a dwell time of 50 ns. It is worth noting that theoretically we could get larger imaging areas with a mosaic image assembled from multiple image tiles; however, the file size of such a large volume EM data set would be huge. For example, an electron microscopic image data set of 1 mm^3^ with 5 nm/pixel lateral resolution, and 25 nm/section would result in a dataset of 1.6 petabytes (PB). Thus, it is necessary to develop an application that can efficiently analyze huge data sets and to have a file server with a sufficiently large storage capacity.

## Carbon Nanotube Tape

Given the practical requirements for automatically collecting sections, collection tapes for serial ultrathin sections should be optimized for the ATUM-SEM method. Tapes for the ATUM must be electrically conductive to capture images without aberrations using SEM, as well as hydrophilic and physically sturdy. Jeff Lichtman and his colleagues at Harvard University, Cambridge, MA, USA, developed a carbon-coated Kapton tape (Hayworth et al., 2014). The Kapton tape is an insulation film, so it lacks conductivity. To make its surface conductive, carbon is deposited on the tape surface using a custom-made high-vacuum carbon-deposition coater with a motorized wheel-to-wheel winder. In addition, since the hydrophobic property of the tape surface causes sections to wrinkle during section collection, it is necessary to hydrophilize the tape surface using plasma discharge (Kubota et al., 2018). Although the carbon-coated Kapton tape is used for section collection with the ATUM (Hayworth et al., 2014; Kasthuri et al., 2015; Morgan et al., 2016), problems remain: the carbon-coated Kapton tape shows a high surface resistance (19–6,530 MΩ/square) and surface inhomogeneities, which can interfere with imaging (Kubota et al., 2018). Moreover, the carbon-coated Kapton tape for the ATUM is yet to be commercialized so the quality of the tape is likely inconsistent and its supply can be sporadic.

Therefore, we searched for substitutes and found a carbon nanotube (CNT)-coated polyethylene terephthalate (PET) tape, with which high-quality electron micrographs of brain tissue can be obtained (Kubota et al., 2018). Positive features of the CNT tape include extremely high conductivity/low surface resistance (242 Ω/square), good resilience, inclusiveness of elements with a very low background signal noise, chemical and mechanical strengths, vacuum compatibility and resistance to beam damage. These features are favorable for ATUM-SEM and, for this application, are comparable or superior to the carbon-coated Kapton tape.

## Optimum Imaging Conditions for SEM

**Figure 2:**
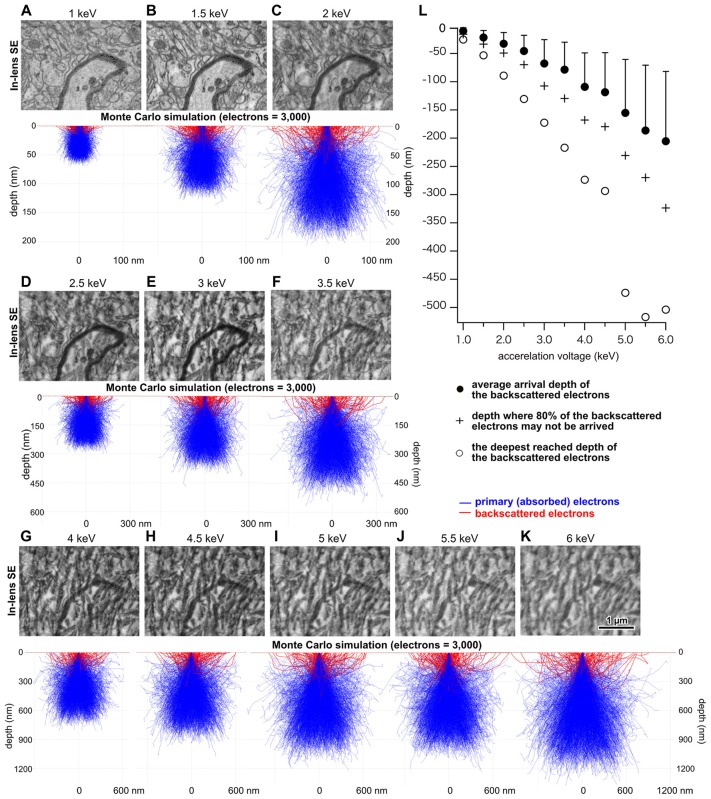
Analysis of projected depth of incident electrons. **(A–K)** Upper panels indicate electron micrographs of brain tissue obtained at various acceleration voltages on an open reel tape. Middle panels show Monte Carlo simulation analysis illustrating potential trajectories of primary and BSEs. **(L)** Depth in the section that was reached by BSEs as a function of acceleration voltage. Adapted from Kubota and Kawaguchi (2018) and Kubota et al. (2018).

Imaging conditions greatly affect the image quality of electron micrographs. The technology to image tissues from ultrathin sections using SEM was invented a decade ago, and it has continued to undergo considerable improvement to this date. There are many factors that affect image quality, including acceleration voltage, probe current, the type of detector for capturing the image signal, aperture, working distance, tissue staining methods, section thickness, etc. It is important to know the optimal value for each imaging factor and to use the best combination thereof. Monte Carlo simulations, which simulate the trajectory of electrons projected onto and into the tissue, are used for determining optimum values (Drouin et al., 2007). However, it is not known whether the Monte Carlo simulation results exhibit actual projected electron trajectories in the plastic embedded brain tissue sections. We examined this issue using electron micrographs captured from 50-nm-thick brain tissue sections embedded in epoxy resin on an open reel tape at various acceleration voltages. We found that as we increased the acceleration voltage, more magnetic crystals of the open reel tape overlapped with the section image (Figure 2; Kubota et al., 2018). We compared this phenomenon with the Monte Carlo simulation analysis to evaluate its validity. We varied the atomic fraction value of the material in the tissue block and found that the actual image results obtained with the open reel tape matched the simulation analysis result well with the atomic fraction value of epoxy resin (nH = 0.53, nC = 0.35, nO = 0.12) without including metals from the staining process (Hennig and Denk, 2007; Kubota et al., 2018). Brain tissue sections are mostly composed of epoxy resin, and stained tissue membranes are just a minor portion of the section. Therefore, we think that the Monte Carlo simulation can adequately describe the actual projected electron trajectories in stained brain tissue sections embedded in epoxy resin.

**Figure 3:**
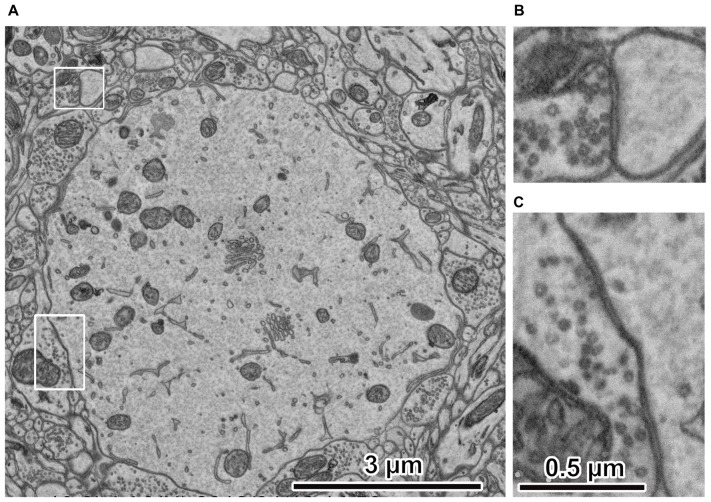
Electron micrographs of rat cerebral cortex. **(A)** Ultrastructure of rat cerebral cortex. The peripheral portion of the cell body is at the center. The electron micrograph was captured using an acceleration voltage 1.5 keV, dwell time 3 μs/pixel, BSE detector, FE-SEM, Regulus 8240 (Hitachi High-Technologies Corp., Tokyo, Japan). **(B)** Spine synapse. An enlarged image in the upper left rectangle in **(A)**. Synaptic vesicles and cleft are clearly observed. **(C)** Somatic synapse. Enlarged image in the lower left rectangle in **(A)**. Synaptic vesicles and clefts are clearly observed. Adapted from Kubota and Kawaguchi (2018) and Kubota et al. (2018).

We found a positive correlation between acceleration voltage and the depth of projected electrons in the tissue, i.e., the interaction volume (Figure 2), which is defined as the volume inside the tissue section in which electrons in the electron beam can interact with tissues. This suggests that a micrograph of a good quality can be obtained with an optimum acceleration voltage, with which the projected electrons interact solely within the section thickness. We assume that signal predominating electrons should be reflected from the more superficial portion of the tissue, because as electrons are projected into the deeper part of the tissue, they must lose energy. Assuming that 80% of the projected electrons carry a sufficient signal that could be detected by the SEM detector, the simulation analysis results suggest that electrons projected with an accelerating voltage of 1.5 keV or 2 keV would interact with the 50-nm-thick tissue section most efficiently (Figure 2) (Kubota et al., 2018). In addition, the detector sensitivity of the recently-developed field emission SEM (FE-SEM) has been significantly improved compared to that in previous years, allowing acquisition of high quality electron micrographs at high magnification using a low acceleration voltage (Kubota et al., 2018). Indeed, an electron micrograph of rat cortex tissues processed with modified rOTO protocol (Hua et al., 2015; Maclachlan et al., 2018) under these conditions shows fine ultrastructure clearly (Figure 3). The Monte Carlo simulation analysis suggests the assumption that the 80% of projected electrons can provide an efficient signal from tissue sections, and their respective interaction volumes in the tissue section could be from the surface to 15 μm depth at an acceleration voltage of 1 keV, to 33 μm depth at 1.5 keV, and to 50 μm at 2 keV (Figure 2). We can use the interaction volume estimation to find optimum imaging conditions, although these estimates could be affected by other mechanical specifications of individual SEMs.

## Automated Serial Block Face Image Acquisition Scanning Electron Microscopy: FIB-SEM

There are several automated image acquisition electron microscopies for obtaining three-dimensional reconstruction at the EM level (Table 1). Each type of microscope has specific features, and understanding these features will help to select the microscopy that suits best for each research project. Of the currently available techniques, the technology of the automated acquisition of serial electron micrographs for three-dimensional reconstruction analysis (3D-EM) has significantly been improved in recent years. This technique has also become easier to use and become more popular among researchers than in the past.

**Table 1:** Typical specifications for currently available automated image acquisition electron microscopies for 3D-electron microscopy (EM).

Section type	Block face		Ultrathin section					
Microscopy	FIB-SEM	SBEM	ATUM-SEM	ATUM-MultiSEM	Wafer SEM	TEMCA	Grid Tape TEM	Grid TEM
Section method	FIB	Ultramicrotome	ATUM	ATUM	Ultramicrotome	Ultramicrotome	ATUM	Ultramicrotome
Section distortion/compression	*52–54°: 78–80%; *90°: no	no	70%–85%	70%–85%	70%–85%	70%–85%	70%–85%	70%–85%
Cutting thickness/*z*-step (nm)	1–1,000	20–100	30–100	30–100	30–100	30–100	30–100	30–100
A hard tissue (ex. Tooth)	OK	difficult	difficult	difficult	difficult	difficult	difficult	difficult
Dwell time (μs)	1–50	0.5–10	0.05–20	0.1–5	0.05–20	~2.7	~2.7	~2.7
Throughput	intermediate	fast	intermediate/fast	extremely fast	intermediate	intermediate/fast	intermediate/fast	intermediate/fast
Charging problem	small	large	small	intermediate	small	small	small	small
Accelaration voltage (keV)	0.5–30	1.5–6	0.5–30	1, 1.5, 2.1, 3	0.5–30	~125	~125	80–125
Alignment	minimal	minimal	necessarily	necessarily	necessarily	necessarily	necessarily	necessarily
Imaging tool	SEM	SEM	SEM	61 channels SEM	SEM	CCD camera	CCD camera	CCD camera
Detector	BSD, SED, ETD	BSD	BSD, SED, ETD	SED	BSD, SED, ETD	CCD	CCD	CCD
Resolution (nm/pixel)	1~	4~	1~	4~	1~	1~	1~	1~
Imaging field of view (μm)	~100	~500	~2,000 or larger	~10,000 or larger	~2,000 or larger	~2,000 or larger	~2,000 or larger	~2,000 or larger

*Angle between two beams. The image distortion is usually scaled to the original block face view image with the focused ion beam-scanning EM (FIB-SEM). BSD, backscattered electron detector; SED, secondary electron detector; ETD, Everhart Thoneley detector. Wafer SEM’ is the method to collect serial sections on a silicon wafer directly and to observe the sections with SEM. Grid Tape transmission EM (TEM)’ is a recent derivative of TEM camera array (TEMCA) using the grid tape with automated tape-collecting ultramicrotomy (ATUM)tome. Grid TEM’ is the method to collect serial sections on grid and to capture the electron micrographs automatically by an image acquisition application with TEM.

FIB-SEM uses two beams: a FIB for milling the block surface, and a scanning electron beam to capture images of the block surface structure. For the FIB, gallium ions are used. The FIB can be extremely narrow in diameter (1–1,000 nm) and sputters a very thin layer from the tissue block surface. It can mill even hard tissues, such as tooth or bone (Tanoue et al., 2018). These features are valuable and unique to the FIB-SEM; the other microscopies cannot achieve such a thin layer cutting nor hard tissue sectioning (Briggman and Bock, 2012; Kubota, 2015). The second beam used in the FIB-SEM is a standard SEM column, and is used for capturing images of the freshly milled block surface after each milling step. There is a limitation in the size of the field of view that can be imaged. The field size is determined by the maximum effective sputtering area for the FIB, which is about 100 μm × 100 μm. Thus, in comparison to the other microscopies, FIB-SEM provides a smaller imaging field of view. A research team working on whole fly brain connectome research in Janelia Research Campus has overcome this limitation with innovative ideas (Hayworth et al., 2015). The researchers split a fly brain into 16 μm-thick tissue slabs with hot knife and reassembled the EM volume data set after the image acquisition (Takemura et al., 2017a, b; Xu et al., 2017). However, there are additional issues with the FIB-SEM.

**Figure 4:**
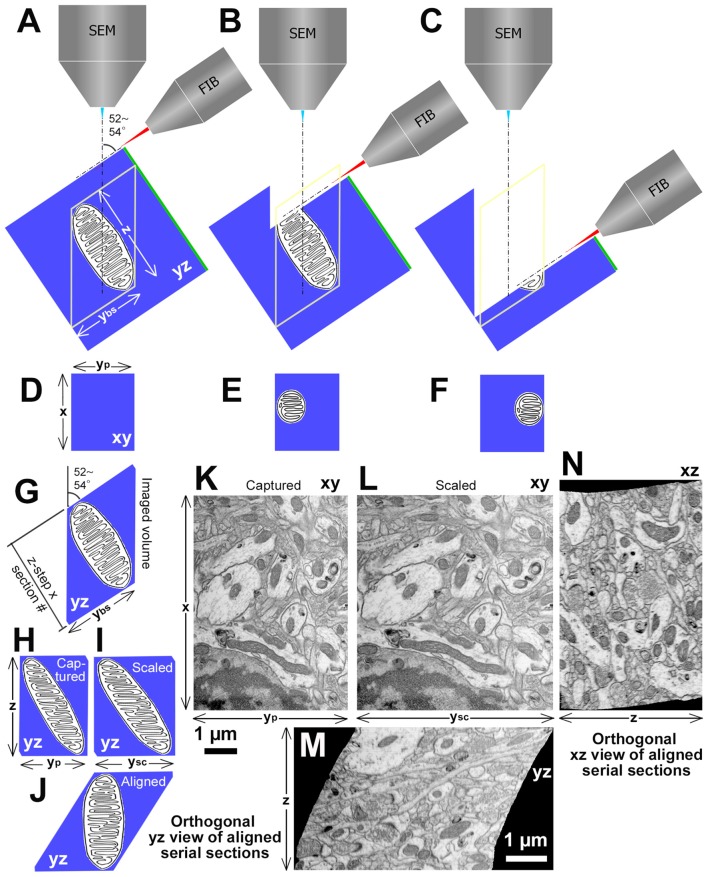
Procedures and examples of volume EM data set obtained with focused ion beam (FIB)-SEM (FIB-SEM) at 52–54° angle between two beams. **(A)** Spatial arrangement of FIB, SEM and a tissue block with a mitochondrion. Green line at one side of the block indicates the surface of the tissue block section for light microscopy. “z” is a depth of the imaged volume and “ybs” is y length of the block surface of the image field. **(B)** An initial part to mill the mitochondrion by FIB. **(C)** Towards the end of milling for the mitochondrion. **(D–F)** Captured images of the mitochondrion on the fresh block surface at each milling step. The mitochondrion location gradually deviates in serial SEM images. “x” is × length of image field and “yp” is y length of the captured image. **(G)** View of the mitochondrion in yz plane. **(H)** Orthogonal yz view of stacked captured serial images. **(I)** Orthogonal yz view of stacked scaled serial images. “ysc” is y length of the scaled image, which equals to the “ybs.” **(J)** Orthogonal yz view of aligned scaled serial images. **(K)** An original electron micrograph, which is the original image of the first section among 600 serial images of rat frontal cortex captured with FIB-SEM (Helios G4, Thermo Fisher Scientific, Waltham, MT, USA) at 2.29 nm/pixel and 7 nm z-step. The scale bar is for horizontal axis. **(L)** A scaled electron micrograph of the image shown in **(K)**. **(M)** Orthogonal yz view of aligned scaled serial images. Please note that the diagram in **(J)** showing the theoretical orthogonal view of a lozenge, which resembles the orthogonal yz view. **(N)** Orthogonal xz view of aligned scaled serial images. The scale bar in **(M)** applies to **(L–N)**, and represents a vertical axis in **(K)**. Modified from Kubota (2015).

First, the projection surface for the FIB must be very smooth without unevenness to capture a high-quality image; otherwise, the acquired image can contain the noise of curtaining effect from FIB milling traces (Liu et al., 2018). Second, it is frequently found that the orthogonal view (yz plane) of the aligned stacking image volume captured with FIB-SEM has a shape of a lozenge, not a rectangle (Figure 4). This is caused by an angle between FIB and SEM beams, typically 52°–54°, which, in turn, sets the SEM beam projection angle relative to the tissue block surface milled by the FIB at 52°–54° (Figures 4A–C). As the serial electron micrographs are actually projected images, these images are distorted with y axis for a factor of 0.786 in the 52° angle from the actual block surface (Figures 4D–F,H,K) taken with FIB-SEM Helios G4 (voxel size: x, 2.29 nm; y, 2.91 nm; z, 2.7 nm; Thermo Fisher Scientific, Waltham, MT, USA). Therefore, captured images need to be scaled in y axis with an appropriate factor (Figures 4H,I,K,L) to restore them to the original size and proportions on the block surface. The scaled serial electron micrographs are aligned using image analysis application (for example, Fiji/Plugins/Registration/Register Virtual Stack Slices). Consequently, the original shape of volume with orthogonally lozenge shape in yz plane should be recovered (Figures 4G,J,M). Note that an elongated object parallel to the block surface gradually deviates from the imaging field of view with serial imaging (Figures 4D–F). This happens only in the yz plane, but not xz plane (Figures 4J,M,N). A FIB-SEM with 90° angle for the two beams captures images without these drawbacks (e.g., NX-9000, Hitachi High-Technologies Corp., Tokyo, Japan). Occasionally, some jitters are found in serial section images before alignment due to the instability of the SEM beam for charging, or deformation/expansion of the block surface by the heat during imaging/sputtering, and this makes the orthogonal xz view slightly irregular (Figure 4N). Despite these issues, the FIB-SEM has unique features that are not available with other methods as discussed in the above, and can be used to capture serial electron micrographs for various research purposes.

## Automated Serial Block Face Image Acquisition Scanning Electron Microscopy: SBEM

SBEM is a well-designed automated serial block face electron micrograph acquisition tool and has several features that make it convenient for neuroscience research. As in FIB-SEM, SBEM uses a scanning electron beam to capture the image of the block surface structure in a chamber, but instead of the FIB, uses an ultramicrotome directly placed in the chamber. A diamond knife is used to cut the block surface, and the surface structure of the tissue block is captured with SEM automatically. Thin sections (~20 nm) can be cut, and imaging throughput is fast (~2 MHz or 0.5 μs/pixel). The imaging area can be larger than with FIB-SEM, because the entire block surface is cut with the diamond knife. SBEM with a custom motorized stage can capture a larger volume data set (~450 μm; Schmidt et al., 2017) than is possible with FIB-SEM. It images the block surface located right under the SEM column, so captured serial images are expected not to be affected by movement. In reality, due to the charging or heating issues, serial images may be found to show jittering, so minimal alignment of serial section images may be necessary.

Several inconvenient issues are encountered with this microscopy. First, section debris falling on the block surface after cutting can obscure ROI during automated imaging occasionally. To address this issue, a new application was developed recently to detect and remove the debris for stable imaging (Titze et al., 2018), which helps with continuous imaging at high quality. Second, electrons impact the block surface, which can cause a crumbling at subsequent sectioning particularly when the dwell time is increased to obtain a better image, even for the tissue block treated with a heavy metal staining protocol for biological tissue samples developed for the SBEM (Deerinck et al., 2010; Ngo et al., 2016; Kubota et al., 2018). An electric charge is easily accumulated at the block surface, because as the diamond knife cuts the block surface, it also removes the silver paste covering the entire block surface that is used for electric to ground. To overcome the drawback, the electron dose (equation below; Kubota et al., 2018) should be kept between 20–40 e-/nm^2^ for satisfactory serial section imaging with smooth ultrathin sectioning (Kubota, 2015). For the imaging condition with 7 nm/pixel, 3 μs/pixel dwell time, and 100 pA beam current, the estimated dose is 38.2 e-/nm^2^, which is within this range, ensuring acquisition of good quality images. Embedding the tissue using epoxy resin mixed with carbon black may reduce the charging issue especially for tissue cells loosely packed in epoxy resin, such as blood cells or yeast (Nguyen et al., 2016). Additional improvement, for example, a detector with better sensitivity or local gas injection to the block surface (Deerinck et al., 2018), can also help to reduce the charging problem.

$$\begin{array}{l} {\text{electron dose (e}}^{-}/{\text{nm}}^{2})\\ \;=\frac{\text{beam current (A) }\times \text{pixel dwell time (s)}}{1.60217657\times 10^{-19}\times {\text{pixel size (nm)}}^{2}} \end{array}$$

## Automated Image Acquisition Electron Microscopy With Serial Ultrathin Sections: TEMCA/TEM

Several microscopies using automated image acquisition of serial ultrathin sections are available. Serial thin sections at ~25 nm thickness can be obtained stably with well polymerized hard epoxy resin embedded tissue. Remarkable features of ultrathin sections in general are that they retain for many years and can be observed many times. As illustrated in Figure 1, these features are conveniently taken advantage of in neuroscience research.

TEMCA is a custom TEM with four 4 MB CCD cameras to obtain images of 4 k × 4 k image size at the bottom of a large scintillator (Bock et al., 2011; Lee et al., 2016). The imaging area can be significantly increased using a mosaic image of many tiles (~450 μm or larger; Lee et al., 2016). This approach is expensive, however, as it requires customized technologies and machines that are yet to be commercially available. TEMCA has been implemented at Harvard, where the TEMCA was originally developed, HHMI Janelia Research Campus, and Allen institute. Recently, a derivative of TEMCA was introduced. Serial ultrathin sections are collected on a grid array tape with ATUM. Electron micrographs of serial sections on a rolled grid tape are obtained with the TEM, which is equipped with a reel to reel motorized system equipped in its specimen chamber (GridStage ReelTM, Voxa, Seattle, WA, USA).

Automated image acquisition of serial ultrathin sections collected on grids with TEM was introduced (Bloss et al., 2018), and the features including the collection of serial sections on a silicon wafer and manual observation with SEM are also available, allowing the acquisition of large volume EM data set using (Boeckeler Instruments, Inc., Tucson, AZ, USA; JEOL Ltd., Akishima, Japan) two commonly available EM tools, namely, ultramicrotome and EM. This likely promotes the use of 3D-EM methods.

## Automated Image Acquisition Electron Microscopy With Serial Ultrathin Sections: Multisem

MultiSEM (Carl Zeiss Microscopy GmbH, Oberkochen, Germany) has 61 channels of SEM, and imaging is very fast (10 MHz or 100 ns/pixel ~) with its in-lens detector (Eberle et al., 2015; Kubota et al., 2018). It can capture brain section prepared by ATUM with a hexagonal field of view of 108 μm × 94 μm at 4 nm/pixel with 61 rectangle tiles of 3,128 × 2,724 pixels size in 1.3 s. The imaging speed is 10^2^–10^3^ times faster than with a regular single beam SEM. For instance, a large volume EM data set of 1 mm cubic brain tissue in this imaging condition with 50 nm thick serial sections can be captured in about 10 h using the MultiSEM, but it takes about a few months to a year using a single beam SEM. There are, however, a few flaws with the MultiSEM. Image resolution is 4 nm/pixel and is about five times larger than single beam SEM (0.7–1.0 nm/pixel). It captures images with the SE detector only, in contrast to the single beam SEM, which uses SE, backscattered electron (BSE) and Everhart Thoneley (ET) detectors. SE detectors may add more noise signals than BSEs do. Consequently, the captured image may show lower contrast. Imaging with the SE detector shows a substantial contaminated area likely as a result of a thin layer of adventitious carbon build up, which results in contamination inside the chamber (Kubota et al., 2018). We assume that those features are part of the reason why the image contrast of electron micrograph obtained with the MultiSEM is somewhat inferior to that of the images captured with a single beam SEM (Figure 3; Kubota et al., 2018). Imaging with MultiSEM requires that the conductive properties of the tape greatly exceed those of a single beam SEM setup (~270 pA: using In-lens SE, 20 μm aperture size, 3 nm/pixel, ~6 keV with Sigma, Carl Zeiss Microscopy, Germany) due to the higher total beam current (61 beams version, 570 pA per beam; 35 nA total). In addition, the stage bias of 28.5 keV is also much higher than in a single beam microscope. It requires a high-quality conductive tape (<100 MΩ/square), such as the CNT tape. Although the MultiSEM requires these ingenious solutions for proper use, it can provide adequate-quality images of a large volume EM data set for neural microcircuit analysis, with extremely high throughput.

## Automated Image Acquisition Electron Microscopy With Serial Ultrathin Sections: Section Compression Problem

It is known that ultrathin sections show a compression artifact from sectioning with ultramicrotome, with compression rate typically in the range of 15%–30% in the cutting direction (Jésior, 1986). This means that ultrathin sections shrink to 70%–85% of the original length depending on cutting conditions. The compression can be reduced by using thicker sections (>30 nm thick), a low angle diamond knife (<30°), or a well polymerized hard epoxy resin; however, changing the cutting speed appears to have no effect (Jésior, 1986). The oscillating diamond knife (DiATOME) can reduce the compression (Studer and Gnäegi, 2000). When grids with thin support membranes are used to collect sections, ultrathin sections may be distorted further during imaging because of the expansion of support membranes by heat from the electron beam, which is usually set at very high energy levels in TEM. Furthermore, the heat can break or destroy the thin supporting membrane, leading to the loss of sections. Fortunately, if serial sections are collected on a rigid silicon wafer or a plastic tape, they are likely to be stable even during imaging with the SEM beam probably without further distortion other than the compression. Therefore, captured images could be scaled to resemble the original shape with adequate compression factor, although compression artifact would not be a problem for investigating synaptic connections in the microcircuit analysis. These features are summarized in Table 1.

## Computer Applications for Three-Dimensional Reconstruction Investigations

The automated applications for registration and segmentation of neuronal elements with high accuracy are useful for processing large-volume data sets. Clay Reid’s group at the Allen Institute, USA introduced their latest research project at YouTube^2^. In this project, the entire neural elements within 100 μm cube of the mouse primary visual cortex were three-dimensionally reconstructed thoroughly for connectomic investigation. *In vivo* calcium imaging analysis of an orientation selectivity in the mouse visual cortex was done by Andreas Tolias at the Baylor College of Medicine, Houston, TX, USA, and the cubic EM volume data set of the cortex was obtained using the TEMCA by Clay Reid at the Allen Institute. Sebastian Seung and his colleagues at Princeton University, Princeton, NJ, USA achieved automated registration and dense segmentation of the large EM data sets using newly-developed computer applications (Lee et al., 2017). This dense segmentation application is based on a deep learning program that requires manually segmented sample EM data sets as ground truth. After tuning for a specific EM data set to be analyzed, they found that automated segmentations were accomplished satisfactorily with only a few mistakes, which were fixed with manual proof reading to establish the completeness of the neural elements reconstructions. The applications are partially made available to the public at Github^3^. This result was achieved for the Machine Intelligence from Cortical Networks program (MICrONS) as a part of the BRAIN Initiative, USA.

The FlyEM team at Janelia Research Campus, USA prepares a *Drosophila’s* whole brain EM data set with 8 nm isometric voxels using FIB-SEM (Takemura et al., 2017a). Computer applications to reconstruct the neural elements three-dimensionally was developed by a research team led by Viren Jain at Google, USA. The accuracy is about the same as those achieved by the Princeton University research team described above, but their approach, “Flood-Filling Networks”, is unique (Januszewski et al., 2018). In addition, the FlyEM team developed a webGL-based viewer, Neuroglancer, for volumetric data analysis to improve viewing performance of large EM images with raster pyramids. These deep learning applications and the Neuroglancer are open-sourced at Github for public use^4^^,^^5^. These pieces of software will undoubtedly advance brain structure analysis in neuroscience.

## Outcomes and Conclusions

Frontier studies with large-volume EM data have already uncovered some previously unknown neural wirings in the brain. Moritz Helmstaedter and his colleagues at Max Plank Institute, Germany described connections among neurons in the rat medial entorhinal cortex using two large volume EM data sets obtained with the SBEM (424 μm × 429 μm × 274 μm size and 183 μm × 137 μm × 158 μm size with the voxel size of 11.24 μm × 11.24 μm × 30 nm; Schmidt et al., 2017). They identified 594 synapses made by 22 excitatory neurons (ExN) following reconstruction that included axons and dendrites. About half of their targets were ExN and the other half were inhibitory non-pyramidal cells (InN). With careful observation of the three-dimensionally reconstructed synaptic contacts among the cells, they concluded that feed forward inhibitory microcircuits are established in the medial entorhinal cortex. Specifically, they showed that pre-synaptic ExNs innervated post-synaptic InNs first along the path of their axons, while innervating post-synaptic ExNs with more distal parts of their axons. Axon fibers of the InNs were frequently myelinated and had larger diameters than those of ExNs, indicating that the conduction velocity of InN axons is faster than that of the axons of presynaptic ExNs. Consequently, it was claimed that the feedforward inhibitory signal could arrive the post-synaptic ExN earlier than the feedforward excitatory signal by simulation.

Nelson Spruston and his colleagues (Bloss et al., 2018) at Janelia Research Campus, Ashburn, VA, USA analyzed the frequency of clustered synapses onto CA1 pyramidal cell dendrites using a large volume EM data set of electron micrographs taken at 3.8 nm × 3.8 nm pixel resolution from 50 nm thick serial thin-sections (x: 200 μm, y: 350 μm, z: 17 μm) containing the stratum radiatum and stratum lacunosum moleculare of the CA1 hippocampus. Ultrathin 50 nm serial sections were collected onto one-hole grids. Image tiles were taken with the TEM and assembled, montaged and aligned using custom alignment software and transformation algorithms in TrakEM2. Dendritic segments of pyramidal cells (*n* = 20), aspiny dendritic segments of putative interneurons (*n* = 5) and excitatory axons (*n* = 43) with their postsynaptic partners were reconstructed manually and analyzed using “Reconstruct” (Fiala, 2005). They found that single presynaptic axons form multiple, spatially clustered inputs onto the distal dendrites in the stratum lacunosum moleculare, but not proximal dendrites in the stratum radiatum, of CA1 pyramidal neurons. These findings enhanced our understanding of the cortical microcircuit structure.

Recent advances in the methodology in the EM and 3-dimensional serial reconstruction of large data sets have made it possible to achieve comprehensive analyses of fine brain structures at individual neuron levels. These new opportunities afford powerful research approaches in neuroscience that were not possible previously. This is an important breakthrough in neuroscience and particularly in the study of brain microcircuitry.

## Ethics Statement

This study was carried out in accordance with the recommendations of “the Guidelines for the Use of Animals” of IBRO and of Animal Care and Use committee of the National Institute for Physiological Sciences. The protocol was approved by the “Animal Care and Use committee.” Every effort was made to minimize animal suffering.

## Author Contributions

YKu conceived the study, designed the experiments, analyzed and interpreted the data shown in the figures, generated the figures and drafted, edited and finalized the manuscript. JS performed the simulation in Figure 2 and edited the manuscript. YKa interpreted the data shown in the figures and edited the manuscript.

## Conflict of Interest Statement

The authors declare that the research was conducted in the absence of any commercial or financial relationships that could be construed as a potential conflict of interest.

## Footnotes

^1^https://imagej.net/TrakEM2

^2^https://www.youtube.com/watch?v=LO8xCLBv6j0&feature=youtu.be

^3^https://github.com/seung-lab

^4^https://github.com/google/ffn

^5^https://github.com/google/neuroglancer

## References

[B1] Bloss E. B. Cembrowski M. S. Karsh B. Colonell J. Fetter R. D. Spruston N. (2018). Single excitatory axons form clustered synapses onto CA1 pyramidal cell dendrites. Nat. Neurosci. 21, 353–363. 10.1038/s41593-018-0084-6 29459763

[B2] Bock D. D. Lee W. C. Kerlin A. M. Andermann M. L. Hood G. Wetzel A. W.. (2011). Network anatomy and *in vivo* physiology of visual cortical neurons. Nature 471, 177–182. 10.1038/nature09802 21390124 PMC3095821

[B3] Bopp R. Holler-Rickauer S. Martin K. A. Schuhknecht G. F. (2017). An ultrastructural study of the thalamic input to layer 4 of primary motor and primary somatosensory cortex in the mouse. J. Neurosci. 37, 2435–2448. 10.1523/JNEUROSCI.2557-16.2017 28137974 PMC6596845

[B4] Bosch C. Martínez A. Masachs N. Teixeira C. M. Fernaud I. Ulloa F.. (2015). FIB/SEM technology and high-throughput 3D reconstruction of dendritic spines and synapses in GFP-labeled adult-generated neurons. Front. Neuroanat. 9:60. 10.3389/fnana.2015.00060 26052271 PMC4440362

[B5] Briggman K. L. Bock D. D. (2012). Volume electron microscopy for neuronal circuit reconstruction. Curr. Opin. Neurobiol. 22, 154–161. 10.1016/j.conb.2011.10.022 22119321

[B6] Bromer C. Bartol T. M. Bowden J. B. Hubbard D. D. Hanka D. C. Gonzalez P. V.. (2018). Long-term potentiation expands information content of hippocampal dentate gyrus synapses. Proc. Natl. Acad. Sci. U S A 115, E2410–E2418. 10.1073/pnas.1716189115 29463730 PMC5877922

[B7] Cardona A. Saalfeld S. Schindelin J. Arganda-Carreras I. Preibisch S. Longair M.. (2012). TrakEM2 software for neural circuit reconstruction. PLoS One 7:e38011. 10.1371/journal.pone.0038011 22723842 PMC3378562

[B8] Deerinck T. J. Bushong E. A. Lev-Ram V. Shu X. Tsien R. Y. Ellisman M. H. (2010). Enhancing serial block-face scanning electron microscopy to enable high resolution 3-D nanohistology of cells and tissues. Microsc. Microanal. 16, 1138–1139. 10.1017/s1431927610055170

[B9] Deerinck T. J. Shone T. M. Bushong E. A. Ramachandra R. Peltier S. T. Ellisman M. H. (2018). High-performance serial block-face SEM of nonconductive biological samples enabled by focal gas injection-based charge compensation. J. Microsc. 270, 142–149. 10.1111/jmi.12667 29194648 PMC5910240

[B10] Denk W. Horstmann H. (2004). Serial block-face scanning electron microscopy to reconstruct three-dimensional tissue nanostructure. PLoS Biol. 2:e329. 10.1371/journal.pbio.0020329 15514700 PMC524270

[B11] Drouin D. Couture A. R. Joly D. Tastet X. Aimez V. Gauvin R. (2007). CASINO V2.48: a fast and easy-to-use modeling tool for scanning electron microscopy and microanalysis users. Scanning 29, 92–101. 10.1002/sca.20000 17455283

[B12] Dufour A. Rollenhagen A. Sätzler K. Lübke J. H. R. (2016). Development of synaptic boutons in layer 4 of the barrel field of the rat somatosensory cortex: a quantitative analysis. Cereb. Cortex 26, 838–854. 10.1093/cercor/bhv270 26574502 PMC4712807

[B13] Eberle A. L. Mikula S. Schalek R. Lichtman J. Knothe Tate M. L. Zeidler D. (2015). High-resolution, high-throughput imaging with a multibeam scanning electron microscope. J. Microsc. 259, 114–120. 10.1111/jmi.12224 25627873 PMC4670696

[B14] Fiala J. C. (2005). Reconstruct: a free editor for serial section microscopy. J. Microsc. 218, 52–61. 10.1111/j.1365-2818.2005.01466.x 15817063

[B15] Harris K. M. Jensen F. E. Tsao B. (1992). Three-dimensional structure of dendritic spines and synapses in rat hippocampus (CA1) at postnatal day 15 and adult ages: implications for the maturation of synaptic physiology and long-term potentiation. J. Neurosci. 12, 2685–2705. 10.1523/JNEUROSCI.12-08-j0001.1992 1613552 PMC6575840

[B16] Hayworth K. J. Kasthuri N. Schalek R. Lichtman J. W. (2006). Automating the collection of ultrathin serial sections for large volume TEM reconstructions. Microsc. Microanal. 13, 86–87. 10.1017/s1431927606066268

[B17] Hayworth K. J. Morgan J. L. Schalek R. Berger D. R. Hildebrand D. G. Lichtman J. W. (2014). Imaging ATUM ultrathin section libraries with WaferMapper: a multi-scale approach to EM reconstruction of neural circuits. Front. Neural Circuits 8:68. 10.3389/fncir.2014.00068 25018701 PMC4073626

[B18] Hayworth K. J. Xu C. S. Lu Z. Knott G. W. Fetter R. D. Tapia J. C.. (2015). Ultrastructurally smooth thick partitioning and volume stitching for large-scale connectomics. Nat. Methods 12, 319–322. 10.1038/nmeth.3292 25686390 PMC4382383

[B19] Helmstaedter M. Briggman K. L. Turaga S. C. Jain V. Seung H. S. Denk W. (2013). Connectomic reconstruction of the inner plexiform layer in the mouse retina. Nature 500, 168–174. 10.1038/nature12346 23925239

[B20] Hennig P. Denk W. (2007). Point-spread functions for backscattered imaging in the scanning electron microscope. J. Appl. Phys. 102:123101 10.1063/1.2817591

[B21] Heymann J. A. Hayles M. Gestmann I. Giannuzzi L. A. Lich B. Subramaniam S. (2006). Site-specific 3D imaging of cells and tissues with a dual beam microscope. J. Struct. Biol. 155, 63–73. 10.1016/j.jsb.2006.03.006 16713294 PMC1647295

[B22] Hua Y. Laserstein P. Helmstaedter M. (2015). Large-volume en-bloc staining for electron microscopy-based connectomics. Nat. Commun. 6:7923. 10.1038/ncomms8923 26235643 PMC4532871

[B23] Januszewski M. Kornfeld J. Li P. H. Pope A. Blakely T. Lindsey L.. (2018). High-precision automated reconstruction of neurons with flood-filling networks. Nat. Methods 15, 605–610. 10.1038/s41592-018-0049-4 30013046

[B24] Jésior J.-C. (1986). How to avoid compression. II. The influence of sectioning conditions. J. Ultrast. Mol. Struct. Res. 95, 210–217. 10.1016/0889-1605(86)90042-x

[B25] Kasthuri N. Hayworth K. J. Berger D. R. Schalek R. L. Conchello J. A. Knowles-Barley S.. (2015). Saturated reconstruction of a volume of neocortex. Cell 162, 648–661. 10.1016/j.cell.2015.06.054 26232230

[B26] Kisvárday Z. F. Gulyas A. Beroukas D. North J. B. Chubb I. W. Somogyi P. (1990). Synapses, axonal and dendritic patterns of GABA-immunoreactive neurons in human cerebral cortex. Brain 113, 793–812. 10.1093/brain/113.3.793 2194628

[B27] Knott G. Marchman H. Wall D. Lich B. (2008). Serial section scanning electron microscopy of adult brain tissue using focused ion beam milling. J. Neurosci. 28, 2959–2964. 10.1523/JNEUROSCI.3189-07.2008 18353998 PMC6670719

[B28] Kubota Y. (2015). New developments in electron microscopy for serial image acquisition of neuronal profiles. Microscopy 64, 27–36. 10.1093/jmicro/dfu111 25564566

[B30] Kubota Y. Hatada S. Kondo S. Karube F. Kawaguchi Y. (2007). Neocortical inhibitory terminals innervate dendritic spines targeted by thalamocortical afferents. J. Neurosci. 27, 1139–1150. 10.1523/JNEUROSCI.3846-06.2007 17267569 PMC6673192

[B31] Kubota Y. Inagaki S. Shimada S. Kito S. Eckenstein F. Tohyama M. (1987). Neostriatal cholinergic neurons receive direct synaptic inputs from dopaminergic axons. Brain Res. 413, 179–184. 10.1016/0006-8993(87)90167-3 2885073

[B32] Kubota Y. Karube F. Nomura M. Gulledge A. T. Mochizuki A. Schertel A.. (2011). Conserved properties of dendritic trees in four cortical interneuron subtypes. Sci. Rep. 1:89. 10.1038/srep00089 22355608 PMC3216575

[B29] Kubota Y. Kawaguchi Y. (2018). “Innovative neural microcircuit analysis using volume electron microscopy and connectome research,” in Jikken Igaku (Experimental Medicine), eds Emoto K. Okabe S. (Tokyo: Yodosha), 158–164.

[B33] Kubota Y. Kondo S. Nomura M. Hatada S. Yamaguchi N. Mohamed A. A.. (2015). Functional effects of distinct innervation styles of pyramidal cells by fast spiking cortical interneurons. Elife 4:e07919. 10.7554/eLife.07919 26142457 PMC4518632

[B34] Kubota Y. Sohn J. Hatada S. Schurr M. Straehle J. Gour A.. (2018). A carbon nanotube tape for serial-section electron microscopy of brain ultrastructure. Nat. Commun. 9:437. 10.1038/s41467-017-02768-7 29382816 PMC5789869

[B35] Kuwajima M. Mendenhall J. M. Lindsey L. F. Harris K. M. (2013). Automated transmission-mode scanning electron microscopy (tSEM) for large volume analysis at nanoscale resolution. PLoS One 8:e59573. 10.1371/journal.pone.0059573 23555711 PMC3608656

[B37] Lee A. W.-C. Bonin V. Reed M. Graham B. J. Hood G. Glattfelder K.. (2016). Anatomy and function of an excitatory network in the visual cortex. Nature 532, 370–374. 10.1038/nature17192 27018655 PMC4844839

[B36] Lee K. Z. J. Li P. Jain V. Seung H. S. (2017). Superhuman accuracy on the SNEMI3D connectomics challenge. bioRxiv

[B38] Liu S. Sun L. Gao J. Li K. (2018). A fast curtain-removal method for 3D FIB-SEM images of heterogeneous minerals. J. Microsc. 272, 3–11. 10.1111/jmi.12723 30098210

[B39] Maclachlan C. Sahlender D. Hayashi S. Molnar Z. Knott G. W. (2018). Block face scanning electron microscopy of fluorescently labeled axons without using near infra-red branding. Front. Neuroanat. [Epub ahead of print]. 10.3389/fnana.2018.00088 PMC6232369 30459565

[B40] Merchán-Pérez A. Rodriguez J. R. Alonso-Nanclares L. Schertel A. Defelipe J. (2009). Counting synapses using FIB/SEM microscopy: a true revolution for ultrastructural volume reconstruction. Front. Neuroanat. 3:18. 10.3389/neuro.05.018.2009 19949485 PMC2784681

[B41] Mikula S. Denk W. (2015). High-resolution whole-brain staining for electron microscopic circuit reconstruction. Nat. Methods 12, 541–546. 10.1038/nmeth.3361 25867849

[B42] Mikuni T. Nishiyama J. Sun Y. Kamasawa N. Yasuda R. (2016). High-throughput, high-resolution mapping of protein localization in mammalian brain by *in vivo* genome editing. Cell 165, 1803–1817. 10.1016/j.cell.2016.04.044 27180908 PMC4912470

[B43] Morales J. Alonso-Nanclares L. Rodríguez J. R. Defelipe J. Rodréguez A. Merchán-Pérez A. (2011). Espina: a tool for the automated segmentation and counting of synapses in large stacks of electron microscopy images. Front. Neuroanat. 5:18. 10.3389/fnana.2011.00018 21633491 PMC3099746

[B44] Morgan J. L. Berger D. R. Wetzel A. W. Lichtman J. W. (2016). The fuzzy logic of network connectivity in mouse visual thalamus. Cell 165, 192–206. 10.1016/j.cell.2016.02.033 27015312 PMC4808248

[B45] Ngo J. T. Adams S. R. Deerinck T. J. Boassa D. Rodriguez-Rivera F. Palida S. F.. (2016). Click-EM for imaging metabolically tagged nonprotein biomolecules. Nat. Chem. Biol. 12, 459–465. 10.1038/nchembio.2076 27110681 PMC4871776

[B46] Nguyen H. B. Thai T. Q. Saitoh S. Wu B. Saitoh Y. Shimo S.. (2016). Conductive resins improve charging and resolution of acquired images in electron microscopic volume imaging. Sci. Rep. 6:23721. 10.1038/srep23721 27020327 PMC4810419

[B47] Ryan K. Lu Z. Meinertzhagen I. A. (2016). The CNS connectome of a tadpole larva of *Ciona intestinalis* (L.) highlights sidedness in the brain of a chordate sibling. Elife 5:e16962. 10.7554/elife.16962 27921996 PMC5140270

[B48] Schindelin J. Arganda-Carreras I. Frise E. Kaynig V. Longair M. Pietzsch T.. (2012). Fiji: an open-source platform for biological-image analysis. Nat. Methods 9, 676–682. 10.1038/nmeth.2019 22743772 PMC3855844

[B49] Schmidt H. Gour A. Straehle J. Boergens K. M. Brecht M. Helmstaedter M. (2017). Axonal synapse sorting in medial entorhinal cortex. Nature 549, 469–475. 10.1038/nature24005 28959971

[B50] Somogyi P. (1977). A specific ‘axo-axonal’ interneuron in the visual cortex of the rat. Brain Res. 136, 345–350. 10.1016/0006-8993(77)90808-3 922488

[B51] Somogyi P. Cowey A. (1981). Combined golgi and electron microscopic study on the synapses formed by double bouquet cells in the visual cortex of the cat and monkey. J. Comp. Neurol. 195, 547–566. 10.1002/cne.901950402 7462443

[B52] Sonomura T. Furuta T. Nakatani I. Yamamoto Y. Unzai T. Matsuda W.. (2013). Correlative analysis of immunoreactivity in confocal laser-scanning microscopy and scanning electron microscopy with focused ion beam milling. Front. Neural Circuits 7:26. 10.3389/fncir.2013.00026 23443927 PMC3581071

[B510] Studer D. Gnäegi H. (2000). Minimal compression of ultrathin sections with use of an oscillating diamond knife. J Microsc. 197, 94–100. 10620152 10.1046/j.1365-2818.2000.00638.x

[B53] Takemura S. Y. Aso Y. Hige T. Wong A. Lu Z. Xu C. S.. (2017a). A connectome of a learning and memory center in the adult *Drosophila* brain. Elife 6:e26975. 10.7554/eLife.26975 28718765 PMC5550281

[B54] Takemura S. Y. Nern A. Chklovskii D. B. Scheffer L. K. Rubin G. M. Meinertzhagen I. A. (2017b). The comprehensive connectome of a neural substrate for ‘ON’ motion detection in *Drosophila*. Elife 6:e24394. 10.7554/elife.24394 28432786 PMC5435463

[B55] Tanoue R. Ohta K. Miyazono Y. Iwanaga J. Koba A. Natori T.. (2018). Three-dimensional ultrastructural analysis of the interface between an implanted demineralised dentin matrix and the surrounding newly formed bone. Sci. Rep. 8:2858. 10.1038/s41598-018-21291-3 29434259 PMC5809602

[B56] Terasaki M. Shemesh T. Kasthuri N. Klemm R. W. Schalek R. Hayworth K. J.. (2013). Stacked endoplasmic reticulum sheets are connected by helicoidal membrane motifs. Cell 154, 285–296. 10.1016/j.cell.2013.06.031 23870120 PMC3767119

[B57] Titze B. Genoud C. Friedrich R. W. (2018). *SBEMimage*: versatile acquisition control software for serial block-face electron microscopy. Front. Neural Circuits 12:54. 10.3389/fncir.2018.00054 30108489 PMC6079252

[B58] Tomassy G. S. Berger D. R. Chen H.-H. Kasthuri N. Hayworth K. J. Vercelli A.. (2014). Distinct profiles of myelin distribution along single axons of pyramidal neurons in the neocortex. Science 344, 319–324. 10.1126/science.1249766 24744380 PMC4122120

[B59] Villa K. L. Berry K. P. Subramanian J. Cha J. W. Oh W. C. Kwon H. B.. (2016). Inhibitory synapses are repeatedly assembled and removed at persistent sites *in vivo*. Neuron 89, 756–769. 10.1016/j.neuron.2016.01.010 26853302 PMC4760889

[B60] White E. L. Amitai Y. Gutnick M. J. (1994). A comparison of synapses onto the somata of intrinsically bursting and regular spiking neurons in layer V of rat SmI cortex. J. Comp. Neurol. 342, 1–14. 10.1002/cne.903420102 8207123

[B61] Xu C. S. Hayworth K. J. Lu Z. Grob P. Hassan A. M. García-Cerdán J. G.. (2017). Enhanced FIB-SEM systems for large-volume 3D imaging. Elife 6:e25916. 10.7554/eLife.25916 28500755 PMC5476429

[B62] Zheng Z. Lauritzen J. S. Perlman E. Robinson C. G. Nichols M. Milkie D.. (2018). A complete electron microscopy volume of the brain of adult *Drosophila melanogaster*. Cell 174, 730.e22–743.e22. 10.1016/j.cell.2018.06.019 30033368 PMC6063995

